# Differences in hospital admissions for acute exacerbations of COPD during the COVID-19 pandemic stratified by stable-state blood eosinophil count

**DOI:** 10.1183/13993003.01125-2023

**Published:** 2023-10-12

**Authors:** Hnin Aung, Hamish McAuley, Kate Porter, Matthew Richardson, Adam Wright, Christopher E. Brightling, Neil J. Greening

**Affiliations:** 1 Department of Respiratory Sciences, University of Leicester, Leicester, UK; 2 Institute for Lung Health, NIHR Leicester BRC, Glenfield Hospital, Leicester, UK

## Abstract

Acute exacerbations of COPD (AECOPD) are driven through different triggers, including infection such as viruses and bacteria. However, nearly 40% of exacerbations are associated with a blood eosinophilia and related to type 2 inflammation (T2-high) [1].

## Tweetable abstract

**Hospital admission for exacerbations of COPD fell only in non-T2-high patients during the COVID-19 pandemic and only in non-eosinophilic admissions. Phenotyping of AECOPD, including at time of exacerbation, is needed for personalised management.** https://bit.ly/3ZiUtYx

*To the Editor:*

Acute exacerbations of COPD (AECOPD) are driven through different triggers, including infection such as viruses and bacteria. However, nearly 40% of exacerbations are associated with a blood eosinophilia and related to type 2 inflammation (T2-high) [1].

The initial year (2020) of the coronavirus disease 2019 (COVID-19) pandemic saw a considerable drop in the prevalence of AECOPD with ≥50% fewer hospitalised exacerbations [2]. In particular, there was a marked decline in infective triggers with a relative reduction of COPD admissions with concomitant pneumonia or influenza of 0.54 and 0.27, respectively, despite more widespread viral PCR testing [2]. This was presumably due to reduced social contact, less travel and the routine wearing of face-masks.

However, considering the heterogeneity behind the pathophysiology of exacerbation and its triggers [3], it is unclear if this pattern observed during the pandemic is still relevant to the exacerbation events associated with eosinophilia or among the population with eosinophilic COPD. This is of importance, as ongoing clinical trials and potential upcoming treatments specifically target COPD patients with a high blood eosinophil count as the therapies are directed towards T2 inflammatory signals [4–6]. In particular, the first biologic trial (BOREAS trial, anti-interleukin (IL)-4/-13) [7] to successfully reach its primary end-point was conducted during the pandemic. This shows great promise, but if eosinophilic exacerbations remained at a similar frequency throughout the pandemic, with a greater proportional reduction in infective triggers, then the effects may be more pronounced.

We hypothesised that hospitalisations for an AECOPD in those with eosinophilia would be less affected by the COVID-19 pandemic, as they are less likely to be driven by microbial infection. Using a previously described cohort of patients with severe COPD we tracked admissions for AECOPD between January 2019 and December 2021, stratified by baseline blood eosinophil count.

Participants were recruited in June 2020 to study the impact of the COVID-19 pandemic on patients with severe COPD, and have been described previously. Briefly, they were eligible if they had a confirmed diagnosis of COPD with airflow obstruction, and under a specialist COPD clinic (Leicester, UK) [8]. Ethics approval was granted by the London-Brent research ethics committee (20/HRA/2510) [9].

Participants were defined as either T2-high or non-T2-high based on the presence of at least one historic blood eosinophil count (BEC) of ≥0.3×10^9^ cells·L^−1^ [10] taken as part of routine care during the year prior to this analysis (*i.e.* 2018). Admission to hospital due to AECOPD during 2019–2021 was captured using electronic patient records. In addition, the highest BEC for each admission was captured, and admissions defined as non-eosinophilic or eosinophilic (cut-off 0.3×10^9^ cells ·L^−1^).

Annualised exacerbation rate (AER) was calculated as the total number of events in each year the participant was in the study. 20 (12.5%) patients died during the study period (n=4 in 2020, n=16 in 2021). A negative binomial mixed model was fitted to compare AER across the years and between groups. Analysis was conducted using glmmTMB package in R [11].

160 patients were included with mean±sd age of 67.3±8.1 years; n=88 (55%) were male. 94% of participants had a Medical Research Council (MRC) dyspnoea score of ≥3, mean±sd forced expiratory volume in 1 s (FEV_1_) 34±13% predicted and 96% were on triple inhaled therapy. No differences other than blood eosinophil count were seen. At least one BEC in 2018 was available for all participants. 56 (35%) subjects met the criteria of T2-high status at baseline and 104 (65%) were non-T2-high. 41 (73%) out of 56 of the T2-high group had two or more exacerbations in the previous year and 20 (36%) out of 56 had been admitted. In the non-T2-high group, 62 (60%) out of 104 had two or more exacerbations in the previous year (between groups p=0.492) and 31 (30%) out of 104 had been admitted (between groups p=0.445). No changes in inhaled prescriptions were made during the study period.

A total of 283 hospitalised AECOPD events were recorded with no difference in T2-high and T2-low over the total year period (120 *versus* 163, p=0.7). A significant reduction in AER was seen in 2020 (p=0.002) compared to 2019. The reduction in admissions was seen in the non-T2-high group, but not the T2-high group (figure 1a) (between groups for 2020 p=0.02). Admission rates returned to similar between groups in 2021 (p=0.44). No difference in length of hospital stay was seen in 2020 compared to 2019 (mean±sd 5.1±6.2 days *versus* 4.7±3.5 days), although a significant increase was seen in 2021 (mean±sd 7.1±7.2 days, p=0.032).

**FIGURE 1:**
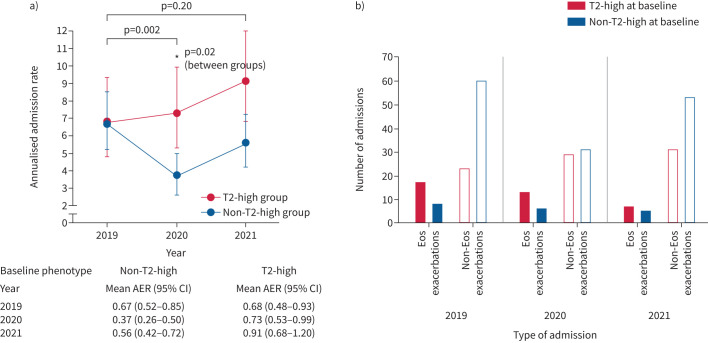
a) Comparison of annualised exacerbation rate (AER) among T2-high and non-T2-high groups over the course of pre- (2019), peak (2020) and late coronavirus disease 2019 pandemic (2021) period. A significant drop in hospitalisations in 2020 was observed, with the drop seen in the non-T2-high population. Data are presented as mean (95% CI). b) Frequency of eosinophilic and non-eosinophilic admissions among the two study groups in each year. Reduction of admissions in the non-T2-high group in 2020 was seen with non-eosinophilic admissions. Eos exacerbation: eosinophilic during acute admission for acute exacerbation of COPD (AECOPD); non-Eos exacerbation: non-eosinophilic during acute admission for AECOPD.

At the time of admission, 227 (80%) of admissions were non-eosinophilic and 56 (20%) were associated with blood eosinophilia. Those in the T2-high group were more likely to account for an admission with a blood eosinophilia (37 (66%) out of 56). Only 19 (8%) out of 227 of admissions in the non-T2-high group had a blood eosinophilia during an admission. In the non-T2-high group, the reduction in hospital admissions during 2020 was only seen in non-eosinophilic admissions, with a similar number of eosinophilic admissions to 2019 and 2021 (figure 1b).

In this cohort of patients with severe COPD, we observed no difference in hospitalisation for AECOPD during 2020, the first year of the COVID-19 pandemic, for patients with T2-high COPD, with the observed reduction in admissions occurring only among the non-T2-high subpopulation. As non-T2-high disease forms two-thirds of our population, an overall significant reduction was seen. Finally, hospital admission rate had returned to pre-pandemic levels in 2021. The non-T2-high population had a ∼50% reduction in hospital admission rate, consistent with other literature [2]. As the non-T2-high disease forms two-thirds of our population, an overall significant reduction was seen. However, to our knowledge, this is the first study to show that hospital admission in the T2-high population remained similar, echoing what a “basal exacerbation rate” without infective triggers may represent [12]. Another study reported an increase in eosinophil count in the pandemic, and is consistent with our findings [13]. Our data highlight the importance of characterisation of patients with COPD, both in the stable state and at the time of an exacerbation, as phenotypes may vary over time and at the time of an event. Treatments that target sub-populations (*e.g.* anti-IL5 or anti-IL-4/-13 biologic therapies) may miss significant signals if exacerbations are all considered similar, highlighting the need for formal assessment of the aetiology of exacerbations in clinical trials. Conversely, periods of changed exacerbation frequency, such as the reduction in infective triggers and reduction in air pollution [14] seen during the pandemic, may result in an accentuated effect, as the remaining exacerbations are more likely to be responsive to targeted intervention. This could have direct implications on the recent findings of dupilumab in eosinophilic COPD [7], conducted during the pandemic, with the future phase 3 trial awaited. Future clinical studies in COPD require to address the individual characteristics of COPD both when stable and during exacerbations.

Our study has limitations. Baseline BEC was taken as part of routine care, and for some participants have been taken following administration of oral corticosteroids, potentially incorrectly assigning them non-T2-high disease. Similarly, some participants may have taken oral corticosteroids prior to hospitalisation or AECOPD blood sampling, which similarly may have lowered BEC during admission. The use of home rescue packs of steroids may have increased during 2020, when virtual management was more common. However, as we saw no reduction in eosinophilic admissions, this seems unlikely. In addition, our population had severe COPD under a specialist service, so may have had increased admissions compared with a less severe disease population. We did not have direct markers of airways inflammatory status, such as sputum eosinophil count. Further mechanistic studies, including sputum inflammometry, are required, to dissect the heterogeneity of each inflammatory event.

In summary, there was no reduction in hospital admissions in patients with severe COPD who had evidence of T2-high disease pre-pandemic. Furthermore, the reduction in admissions was seen in non-eosinophilic admissions in the non-T2-high population. These data support a personalised management approach at the time of an exacerbation.

## Shareable PDF

10.1183/13993003.01125-2023.Shareable1 This one-page PDF can be shared freely online.
Shareable PDF ERJ-01125-2023.Shareable
