# Supplementary material for: Differences in hospital admissions for acute exacerbations of COPD during the COVID-19 pandemic stratified by stable-state blood eosinophil count
Source: Eur Respir J. 2023 Oct 12;62(4):2301125. doi: 10.1183/13993003.01125-2023 (PMC10568037; doi:10.1183/13993003.01125-2023)
Supplement: Supplementary file 1 [file ERJ-01125-2023.Shareable.pdf]

SHAREABLE PDF

Differences in hospital admissions for acute exacerbations of COPD during the COVID-19 pandemic stratified by stable-state blood eosinophil count

Hnin Aung[1,2], Hamish McAuley[1,2], Kate Porter[2], Matthew Richardson[1,2], Adam Wright[1,2], Christopher E. Brightling[1,2] and Neil J. Greening[1,2]

[1]Department of Respiratory Sciences, University of Leicester, Leicester, UK. [2]Institute for Lung Health, NIHR Leicester BRC, Glenfield Hospital, Leicester, UK.

Corresponding author: Neil J. Greening (neil.greening@leicester.ac.uk)

Shareable abstract (@ERSpublications)
Hospital admission for exacerbations of COPD fell only in non-T2-high patients during the COVID-19 pandemic and only in non-eosinophilic admissions. Phenotyping of AECOPD, including at time of exacerbation, is needed for personalised management. https://bit.ly/3ZiUtYx

Cite this article as: Aung H, McAuley H, Porter K, *et al*. Differences in hospital admissions for acute exacerbations of COPD during the COVID-19 pandemic stratified by stable-state blood eosinophil count. *Eur Respir J* 2023; 62: 2301125 [DOI: 10.1183/13993003.01125-2023].

This extracted version can be shared freely online.

Copyright ©The authors 2023.

This version is distributed under the terms of the Creative Commons Attribution Licence 4.0.

Received: 3 July 2023
Accepted: 7 Sept 2023

To the Editor:

Acute exacerbations of COPD (AECOPD) are driven through different triggers, including infection such as viruses and bacteria. However, nearly 40% of exacerbations are associated with a blood eosinophilia and related to type 2 inflammation (T2-high) [1].
